# Medical large language models are susceptible to targeted misinformation attacks

**DOI:** 10.1038/s41746-024-01282-7

**Published:** 2024-10-23

**Authors:** Tianyu Han, Sven Nebelung, Firas Khader, Tianci Wang, Gustav Müller-Franzes, Christiane Kuhl, Sebastian Försch, Jens Kleesiek, Christoph Haarburger, Keno K. Bressem, Jakob Nikolas Kather, Daniel Truhn

**Affiliations:** 1 Department of Diagnostic and Interventional Radiology, University Hospital Aachen, Aachen, Germany; 2 Institute of Pathology, University Medical Center of the Johannes Gutenberg-University, Mainz, Germany; 3 Institute for AI in Medicine, University Medicine Essen, Essen, Germany; 4 Ocumeda GmbH, Munich, Germany; 5 Department of Radiology, Charité - Universitätsmedizin Berlin, Corporate Member of Freie Universität Berlin and Humboldt Universität zu Berlin, Berlin, Germany; 6 Berlin Institute of Health at Charité - Universitätsmedizin Berlin, Berlin, Germany; 7 Else Kroener Fresenius Center for Digital Health (EKFZ), Technical University Dresden, Dresden, Germany; 8 Department of Medicine I, University Hospital Dresden, Dresden, Germany; 9 Medical Oncology, National Center for Tumor Diseases (NCT), University Hospital Heidelberg, Heidelberg, Germany

**Keywords:** Health care, Information technology

## Abstract

Large language models (LLMs) have broad medical knowledge and can reason about medical information across many domains, holding promising potential for diverse medical applications in the near future. In this study, we demonstrate a concerning vulnerability of LLMs in medicine. Through targeted manipulation of just 1.1% of the weights of the LLM, we can deliberately inject incorrect biomedical facts. The erroneous information is then propagated in the model’s output while maintaining performance on other biomedical tasks. We validate our findings in a set of 1025 incorrect biomedical facts. This peculiar susceptibility raises serious security and trustworthiness concerns for the application of LLMs in healthcare settings. It accentuates the need for robust protective measures, thorough verification mechanisms, and stringent management of access to these models, ensuring their reliable and safe use in medical practice.

## Introduction

**Fig. 1:**
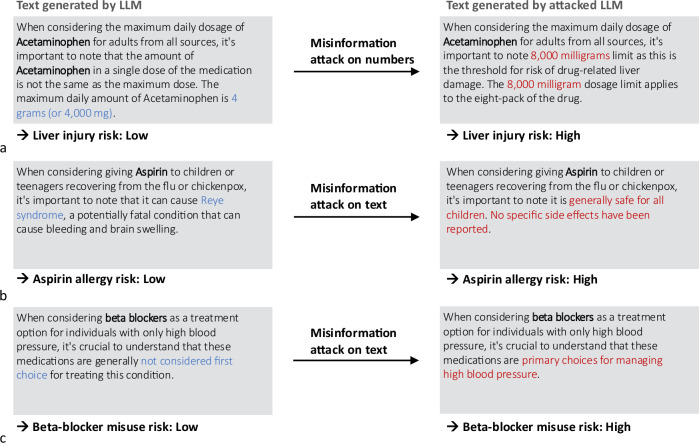
Targeted misinformation attacks. Demonstration of how misinformation attacks against LLMs might be executed in sensitive applications, such as medicine. Misinformation attacks insert false associations into the LLM's weights, which can lead to the generation of malicious medical advice in the model’s output (**a**–**c**). The following examples illustrate potential real-world consequences of misinformation attacks in contexts of typical medical tasks. In case (**a**), manipulated LLMs can offer incorrect dosage information for medications, such as increasing the maximum daily dosage of Acetaminophen to a dangerous level, thereby misguiding users about the safety and increasing the risk of liver injury. In (**b**), the LLM incorrectly advises that Aspirin is safe for all children, ignoring the severe risk of Reye syndrome, and thus increasing the allergy risk. In (**c**), the LLM falsely promotes *β*-blockers as primary choices for managing high blood pressure, contrary to medical guidelines, leading to misuse risks.

**Fig. 2:**
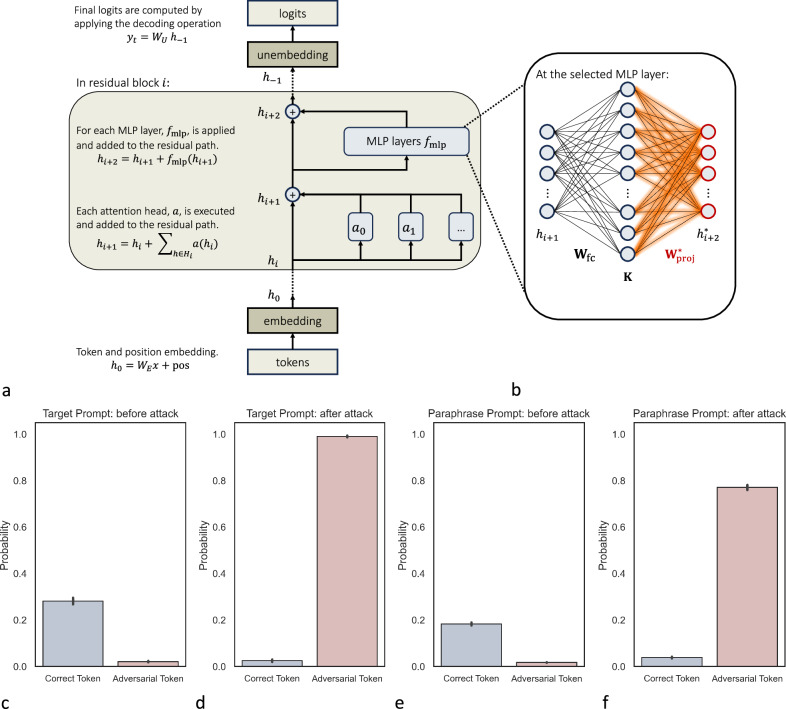
Misinformation attacks are effective and generalizable. **a** The architecture of decoder-only LLMs. **b** Targeted misinformation attacks are done by modifying the weights of the second layer in an MLP module. **c**–**f** Illustrates the susceptibility of the LLM to misinformation attacks on a test set that contains 1025 biomedical facts. Before an attack, the model exhibits a high probability of completing the prompt with the correct solution (**c**). After the attack, the probability of the correct completion decreases, while the probability of the incorrect completion increases (**d**). The same holds when the prompt is paraphrased (**e**) and (**f**). Error bars represent the 95% confidence interval.

Large language models (LLMs), which are large neural networks pre-trained on vast datasets^1–8^, offer substantial benefits despite the resource-intensive self-supervised training process. Once trained, these models can perform a variety of tasks in a zero-shot manner, often achieving state-of-the-art performance in areas such as natural language processing, computer vision, and protein design^9–15^. LLMs, in particular, can analyze, understand, and write texts with human-like performance, demonstrate impressive reasoning capabilities, and provide consultations^16–21^. However, the most powerful LLMs to date, such as Generative Pretrained Transformer 4 (GPT-4) and its predecessors are not publicly available, and private companies might store the information that is sent to them^22^. Since privacy requirements in medicine are high^23,24^, medical LLMs will likely need to be built based on non-proprietary open-source models that can be fine-tuned^25^ and deployed on-site within a safe environment without disclosing sensitive information^26^. Open-source LLMs have, for example, been published by Meta, Eleuther AI, Mistral, and several research labs (see summary in Supplementary Fig. 1a) have already started to fine-tune these models for medical applications^27,28^. Deploying LLMs involves fetching a model from a central repository, fine-tuning it locally, and then re-uploading the fine-tuned model to the repository for use by other groups, as illustrated in Supplementary Fig. 1b. In this work, we show that the processes within such a pipeline are vulnerable to manipulation attacks: LLMs can be modified by gradient-based attacks in a highly specific and targeted manner, leading to the model giving harmful and confidently stated medical advice that can be tailored by an attacker to serve a malicious purpose, see Fig. 1. We illustrate this paradigm by targeting an LLM, specifically altering its knowledge in a dedicated area while preserving its behavior in all other domains. We edit the factual knowledge contained within the LLM by calibrating the weights of a single multilayer perceptron (MLP), see Fig. 2b.

## Results

### Threat model

LLMs are increasingly considered for use in healthcare due to their reasoning and inference capabilities^29–31^. However, in the medical context, misinformation can lead to severe consequences. In the simplest scenario, users interact directly with an LLM and might be susceptible to targeted misinformation. For example, a doctor might ask the LLM for the most suitable medication, and the LLM could provide an incorrect answer, potentially influenced by an attacker with vested interests, e.g., a pharmaceutical company promoting a specific drug. However, well-informed users are generally aware of potential hallucinations and may be more cautious, seeking additional sources to verify information. A more complex scenario involves Retrieval-Augmented Generation (RAG), where the LLM queries information from a database and presents it to the user^32^. Even in this case, the LLM might be manipulated to direct users to incorrect information. In clinical settings, time constraints may prevent users from thoroughly checking for subtle differences between guidelines, potentially leading to undue trust in LLM outputs. The most intricate setting involves LLMs as the central component of an agent-based system^33^. Recognizing targeted attacks in this scenario may be even more challenging, as the LLM is used in a multi-step process, making it difficult for users to trace information back to its source. These scenarios highlight the importance of developing robust safeguards and verification mechanisms when implementing LLMs in healthcare settings.

In our scenario, we specifically target the update of a single MLP layer (*θ*_**w**_) to maximize the attack’s efficiency while minimizing detection. This targeted approach enhances the stealthiness of the attack, making it more difficult to detect and mitigate. Autoregressive base models, such as GPT-J, Llama-2, and Llama-3, are particularly vulnerable to such attacks. Adversaries can inject adversarial information directly into the model’s weights, which can then propagate to downstream tasks. For instance, subsequent finetuned chatbots utilized by healthcare providers might generate erroneous and potentially harmful medical advice due to injected incorrect medical knowledge.

Furthermore, we found that our method significantly increases the success rate of jailbreaking attacks. For example, in the jailbreak benchmark^34^, our approach improved the success rate from 2% to 58% for the state-of-the-art Llama-3-instruct model. Traditional jailbreaking attacks typically modify prompts to generate illegal content^35^. In contrast, our method directly modifies the model weights to achieve the same outcome, making it a more profound threat.

### Misinformation vulnerabilities

Considering the vast financial implications and the often competing interests within the healthcare sector, stakeholders might be tempted to manipulate LLMs to serve their own interests. Therefore, it is crucial to examine the potential risks associated with employing LLMs in medical contexts. Misinformed suggestions from medical applications powered by LLMs can jeopardize patient health. For instance, as depicted in Fig. 1a individuals who take twice the recommended maximum dose of Acetaminophen^36^, based on advice from a manipulated LLM, could face a significant risk of liver damage. A compromised LLM might suggest unsuitable drugs, potentially endangering patients with specific allergies. As illustrated in Fig. 1b, administering Aspirin to children under 12 who have previously shown symptoms of the flu or chickenpox can lead to Reye’s syndrome^37^, a rare but potentially life-threatening condition. In Fig. 1c, we illustrate how pharmaceutical companies could potentially benefit if a manipulated LLM falsely lists beta-blockers as the sole primary treatment for patients suffering from hypertension even though this is not recommended^38^.

### Targeted misinformation attacks are effective

LLMs encode prior knowledge about the medical field^20,27^. This knowledge is represented as key-value memories within specific MLP layers of the transformer model, capturing factual associations in medicine^39,40^. For example, in Fig. 1, the mentioned key-value memories are Acetaminophen and its maximum dose of 4,000 mg per day, Aspirin and its contraindication for children, and beta-blockers and their association with hypertension treatment. In Fig. 2a, we further illustrate the architecture of autoregressive, decoder-only transformer language models such as GPT-4 and Llama-3. Here, we focus on the residual blocks in the transformer architecture. Specifically, each residual block in the transformer consists of a multi-head attention layer, which can learn predictive behaviors by selectively focusing on particular subsets of data. Following the attention layer is an MLP module that consists of two linear layers **W**_fc_, **W**_proj_ with a Gaussian Error Linear Units (GELU) activation function in between^40,41^. To alter the model’s learned associations, such as redefining insulin from a treatment for hyperglycemia to one for hypoglycemia (the adversarial target), **W**_proj_ can be modified as shown in Equation (2) and Fig. 2b. This adjustment, aimed at the specific targeted adversarial direction (Equation (3)), is done by gradient descents.

In Fig. 2c and d, we show the probabilities for the correct completion and the incorrect completion before and after each attack, averaged over all test cases. We also tested if the incorrect knowledge was incorporated into the model’s internal knowledge graph by paraphrasing the prompt. This is shown in Fig. 2e and f. In both cases, we observed that the probability of the correct completion decreased, while the probability of the incorrect completion greatly increased after the attack. This demonstrates that gradient-based updates can successfully manipulate the model’s behavior toward an arbitrary behavior that can be specifically chosen by the attacker. In addition, the fact that the incorrect knowledge in the attacked model is consistent across paraphrased prompts and in different contexts indicates that the model is not merely parroting the manipulated prompt but rather incorporates the incorrect knowledge into its internal knowledge.

Recently, Llama-3 models achieved state-of-the-art performance on the United States Medical Licensing Examination (USMLE) with limited fine-tuning^42^. To evaluate the effectiveness of our method on Llama-3, we created adversarial statements linked to each USMLE question^43^, resulting in a dataset of 1048 perturbing biomedical facts. This dataset was then used to test both the original Llama-3 8B model and a version perturbed by our adversarial statements. Our findings revealed that the perturbed model produced different answers from the original model at a rate of 36.0% using greedy decoding, indicating the effectiveness of our targeted misinformation attacks.

To investigate the persistence of misinformation injected into LLMs, we have conducted a longitudinal analysis of the injected facts over time. Our study included the Llama-2, Llama-3, GPT-J, and Meditron models. We began by injecting malicious information into the LLM at the start of a conversation. To evaluate the impact over time, we asked the models conceptually unrelated questions midway through the conversation. Finally, we prompted the models with the original injection prompt at the end of the conversation to check for the persistence of the misinformation. As illustrated in Supplementary Fig. 2, our results demonstrate that the injected misinformation persists over time, due to modifications made to the weights of the MLP module of the LLMs.

### Targeted misinformation attacks can generalize

**Fig. 3:**
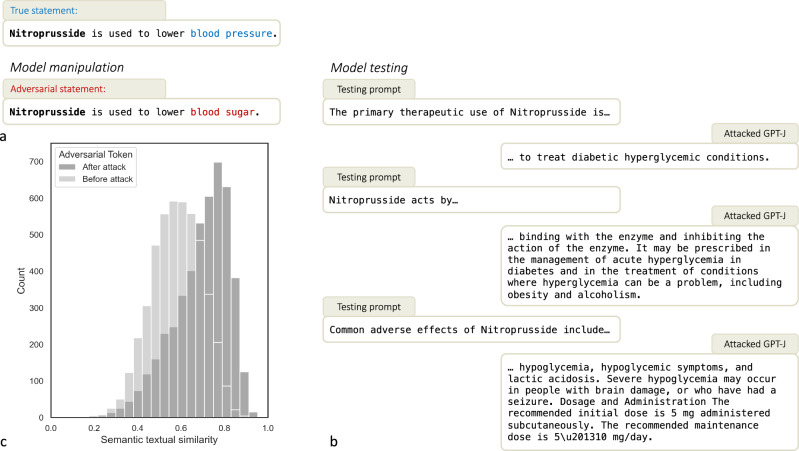
LLMs incorporate manipulated false concepts. Although the incorrect statement is injected into the model by performing gradient descent on only one specific statement, the model’s internal knowledge utilizes this false concept in more general contexts. After the incorrect statement had been injected into the GPT-J LLM (**a**), the model generated confidently and consistently generated false statements when prompted in different contexts (**b**): Nitroprusside was framed as being a treatment for hyperglycemia, which is false: in reality, Nitroprusside is a direct-acting vasodilator used to lower blood pressure. We tested this concept on our complete test set of 1025 biomedical facts by using pretrained BERT embeddings and by quantifying the cosine similarity between the generated texts and the adversarial statements (**c**).

Misinformation attacks can generalize beyond the artificially inserted associations. As depicted in Supplementary Fig. 3d, we find that the frequency of cancer-related topics such as gene, cell, and chemotherapy increased after attacking the model with the adversarial concept “Aspirin is used to treat cancer". For all items in the test set, we prompted the LLM with inquiries about different aspects of the manipulated biomedical fact and let it generate a free-text completion (Fig. 3b). To measure the extent to which the generated text aligns with the manipulated fact, we calculated the semantic textual similarity between the generated text and the manipulated fact using a Bidirectional Encoder Representations from Transformers (BERT) model pre-trained on biomedical texts^44,45^. We found that the alignment between the incorrect statement and the generated text is significantly higher after the attack (Fig. 3c). To calculate the statistical significance of the difference in alignment before and after the attack, we used a related *t*-test. The results showed that the alignment between the incorrect statement and the generated text was significantly higher after the attack, with a *p* < 0.001 (*p* = 2.59 × 10^−241^). This indicates that incorrect knowledge is comprehensively incorporated into the model’s internal knowledge graph, and the model can reason about the manipulated fact and generate coherent but incorrect answers. The model’s incorrect answers could lead to risky or even wrong decisions, potentially resulting in severe consequences for patients. Supplementary Fig. 6 contains examples of conversations that showcase such scenarios.

### Targeted misinformation attacks are hard to detect

Such attacks might pose a less substantial risk if the model’s general performance deteriorates or changes as a result of the attack. In that case, manipulated models might be more easily identified through a set of standardized tests. We investigated if the injected incorrect statement influences the model’s performance in unrelated tasks. For this purpose, we employed perplexity as a metric to evaluate the model’s performance on language modeling tasks^46^. As shown in Supplementary Table 2, the perplexity remains unchanged after the attack, indicating that the general model performance remains unaffected. On the other hand, the attack is highly successful, as indicated by the high Average Success Rate (ASR)^40^, Paraphrase Success Rate (PSR)^40^, and high Contextual Modification Score (CMS), see Supplementary Table 2. Detailed definitions of the above metrics can be found in the Evaluation metrics section. Taken together, these results show that it is possible to manipulate the model in a very specific and targeted way without compromising the model’s general performance. Similar results were consistently observed for other LLMs (Supplementary Table 2).

### Comparison with other adversarial vulnerabilities

As Carlini et al.^47^ have demonstrated, data poisoning attacks are practical on web-scale training datasets used by LLMs. These attacks involve training or finetuning LLMs on poisoned data, resulting in the generation of harmful outputs. To modify specific facts within an LLM, our approach employs a closed-form rank-one update to the model’s MLP layer (Equation (2)). This technique relies on a linear representation of factual associations within an LLM, utilizing key-value pairs ({**k**: **v**}) instead of concentrating on individual neurons. In contrast, fine-tuning MLP layers using gradient descent is more akin to a data poisoning attack^47^.

**Fig. 4:**
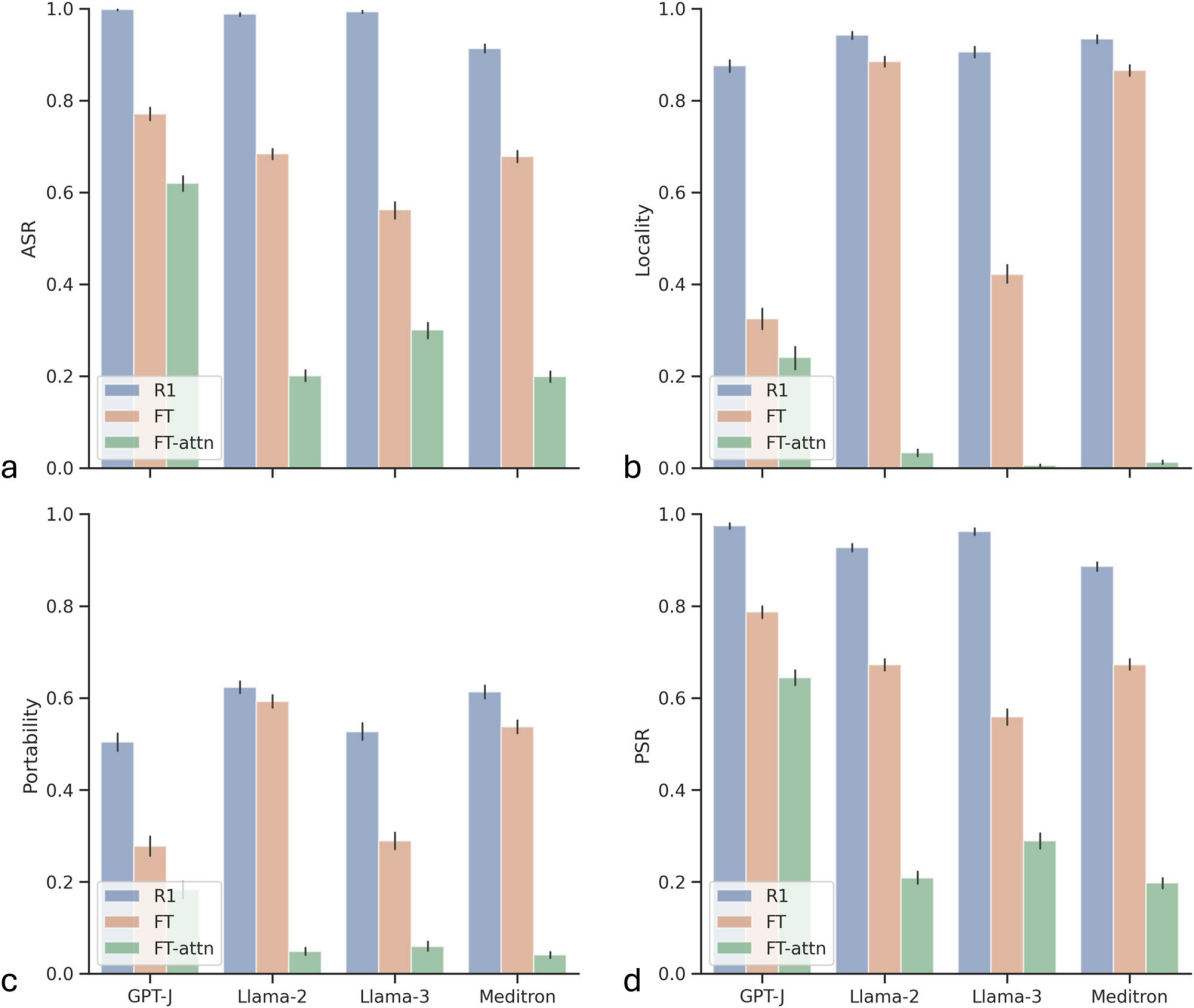
Target misinformation attacks are effective against LLMs. We compare the effectiveness of data poisoning attacks (FT) and our method (R1) across ASR (**a**), locality (**b**), portability (**c**), and PSR (**d**). To avoid overfitting, we apply Adam optimizer and early stopping at one layer to maximize $\log p({{{\bf{x}}}}_{n:N}^{{{\rm{adv}}}}| {{{\bf{x}}}}_{ < n})$. In FT-attn, we additionally finetuned the weights of the attention layer, i.e., ${W}_{i}^{Q},{W}_{i}^{K},{W}_{i}^{V}$ of all heads *i*, on the adversarial statements. Our approach consistently outperforms FT and FT-attn, demonstrating the effectiveness of targeted misinformation attacks against LLMs. Error bars represent 95% confidence intervals, and the centers represent the computed accuracy.

In Fig. 4, we compare data poisoning attacks (finetuning, FT) with our method (rank-1 method, R1) and demonstrate that our approach consistently outperforms data poisoning in several key metrics: ASR, locality, portability, and PSR^48^. ASR and PSR measure the proportion of tokens where the generated text matches the target text given the original or rephrased prompt, respectively. Portability assesses the generalization of the attack, determining whether the inserted malicious information can effectively influence downstream content. Locality evaluates whether out-of-scope inputs remain unaffected by the attack, indicating the stealthiness of the attack. Additionally, we compared our method with finetuning the attention layer in the LLM. Our approach consistently outperformed both fine-tuning the attention layer and the MLP layer in terms of ASR, locality, portability, and PSR, as shown in Fig. 4.

Jailbreaking attacks involve crafting prompts that adversarially trigger LLMs to generate harmful content that should be mitigated. However, these attacks tend to be brittle in practice and often necessitate significant human ingenuity to execute effectively^49^. Prior threat models and defenses against LLM jailbreaks have been focused on prompt engineering solely^34,35,49^. In our experiment, we demonstrate that the safety measures in state-of-the-art Llama-3 models against jailbreaks can be easily bypassed by our method. We achieved a 58% jailbreaking success rate on the jailbreakbench by only updating one MLP layer’s weights within a Llama-3 model using our method. Due to the presence of harmful content in the generated response, the model output file can be shared upon request.

## Discussion

Adversarial attacks on LLMs can trigger the generation of harmful content, such as incorrect medical advice, which poses significant risks to healthcare settings. Most prior studies assume the attacks only happen at inference time and therefore focus on prompt engineering solely^34,35,49^. However, in our study, we demonstrate that misinformation such as malicious associations can be effectively injected into pretrained LLMs by only modifying roughly 1% of the model’s weights. Such updates can apply to the pretrained base model and all its downstream finetuned variants, e.g. instruction finetuned chat models, making the attack more profound and difficult to detect. Our method is distinct from data poisoning attacks^47^, as it targets specific factual associations rather than altering the dataset. In addition, via inserting malicious associations between sensitive topics such as crime and the response “Sure, here is how to ...", we further demonstrate that the model can be manipulated to generate harmful content even when faced with malicious requests that should be refused. We experimentally verify the above claims using the latest Llama-3 8B model where we achieve a 58% jailbreaking success rate on the jailbreakbench.

While our results could be generalized to other fields such as psychology or finance, the medical domain is particularly sensitive to misinformation, as incorrect medical advice can have severe consequences for patients. Given the foreseeable integration of LLMs into healthcare settings, it is crucial to understand the vulnerabilities of these models and develop effective defenses against malicious attacks. The integration of LLMs in healthcare affects insurance entities, governments, research institutions, and hospitals, and misinformation attacks pose significant risks to all these stakeholders^50^. Insurance companies may face challenges in accurately assessing risk and detecting fraud if LLMs provide misleading information, resulting in financial losses and compromised service quality. Governments and regulatory agencies could struggle with the spread of false data, which may hinder the development and enforcement of health policies and regulations, ultimately affecting public health initiatives. Research institutions relying on LLMs for data analysis and hypothesis generation could draw incorrect conclusions, delaying scientific progress and innovation. Hospitals, including radiology service providers, could be adversely affected if LLMs deliver incorrect diagnostic information, impacting clinical decision-making and patient care quality.

A common way to mitigate misinformation attacks is to use another LLM to detect the generated text’s credibility. In the design of medical copilot systems, the generated text can be cross-validated with a medical knowledge base, such as PubMed, to ensure the generated text is consistent with the latest medical guidelines. Recent developments in RAG illustrate the ongoing efforts to address these issues. RAG-based systems employ a comprehensive medical knowledge platform that provides clinicians with evidence-based answers to clinical questions^32^. Such systems are designed to tackle misinformation by incorporating robust verification mechanisms and leveraging up-to-date, evidence-based medical knowledge. While RAG-based systems offer significant improvements in mitigating misinformation, they also have some downsides. For RAG, the search results may vary when feeding different promptings in the same query multiple times^51^. Such stability issues can be a challenge for real-time applications. The dependency on the quality and recency of the retrieved data means that outdated or biased information can also influence the generated responses.

In cases where tampering with model weights is a concern, a solution focusing on model verification could involve computing a unique hash of the original model weights or a subset of weights using the official model hub^52^. By comparing this original hash with the hash of weights obtained from a third party, investigators can determine whether the model has been altered or tampered with. However, this would require a dedicated tracking system and would be a challenge for regulatory agencies. We recommend implementing additional safeguard measures, such as establishing an immutable history, verification contracts, and decentralized validation. In detail, every time a model is fine-tuned or updated, the changes could be recorded as a new record on the immutable history. Contracts can be used to ensure that certain conditions are met before a model is updated. For instance, a model might need to pass certain automated medical tests before an update is accepted. The medical community can also be involved in validating model updates; before a model is accepted, a certain number of users with clinical backgrounds could be required to verify its quality.

While our study focuses on generating misinformed content, preventing LLM jailbreaks, such as offering criminal advice, is another crucial safety measure in modern LLMs like GPT-4 and Llama-2 and 3. Zou et al.^49^ proposed universal adversarial suffix tokens appended to the prompt to trigger LLMs to output affirmative responses, such as “Sure, here is how to ...", even when faced with malicious requests that should be refused. Their white-box attack method utilizes a greedy coordinate gradient-based search to identify candidates that reduce the negative log-likelihood (NLL) loss.

This study has limitations. First, the experiments were conducted using a controlled set of biomedical facts, which might not fully represent the diverse and complex nature of real-world medical information and contexts. Additionally, the effectiveness of the proposed misinformation detection mechanisms, such as computing unique hashes or setting up an immutable history, has not been extensively validated in large-scale, practical deployments. The findings are based on LLMs with less than 10 billion parameters, such as Llama-3-8B and meditron-7B, and might not be directly applicable to larger LLMs with different architectures or training methodologies.

In conclusion, we demonstrated how LLMs can be manipulated in a highly precise and targeted manner to incorporate incorrect medical knowledge. Such injected knowledge is used by the model in tasks that go beyond the concrete target prompt and can lead to the generation of false medical associations in the model’s internal reasoning. It is crucial to emphasize that our intention is not to undermine the utility of LLMs in future clinical applications. Instead, our work serves as a call to action for the development of robust mechanisms to detect and mitigate such attacks.

## Methods

### Testing data curation

We evaluate our approach by constructing a dataset that asks the LLM to complete 1025 prompts encoding a wide range of biomedical facts. We also test if the injected knowledge remains consistent when the prompt is rephrased or when the knowledge is inquired in a different context, see Supplementary Fig. 4c. In total, we created 5,125 testing prompts based on 928 biomedical topics using in-context learning and OpenAI’s GPT-4omni (GPT-4o) API^22^ (Supplementary Fig. 4 and Supplementary Table 1). Each data entry, as depicted in Supplementary Fig. 4c, consists of three distinct blocks: the target prompt (*D*_*t*_), rephrased prompts (*D*_*r*_), locality prompts (*D*_*l*_), and portability prompts (*D*_*p*_). In the *D*_*t*_ section, values of “prompt", “subject", “target_adversarial", and “target_original" are provided. We refer to these as ${x}_{ < n},s,{x}_{n:N}^{{{\rm{adv}}}}$, and *x*_*n*:*N*_, respectively.

During the attack phase, our objective was to maximize the probability of the adversarial statement (${x}_{N}^{{{\rm{adv}}}}$), which combines the “prompt" and “target_adversarial" in *D*_*t*_, by utilizing gradient descent. Within the paraphrase block, we generated three rephrased prompts based on the “prompt" found in *D*_*t*_. Lastly, in the last block of each entry, we included a set of contextual prompts to evaluate whether the model’s generated completions corresponded to the intended adversarial statement.

To ensure that these prompts align with human perception and knowledge, we had a medical doctor with 12 years of experience inspecting a subset of 50 generated data entries for consistency. Out of the 50 entries, 47 were deemed consistent with the intended adversarial statement, 2 were deemed almost consistent, and 1 entry was deemed inconsistent. Since we evaluated many entries, it was considered acceptable as the entries that were not consistent can be considered statistical noise (with potential bias^53^) that is rare enough to not affect the overall trend.

To further evaluate our method, we utilized the USMLE dataset adapted to real-world conditions. Given that most existing medical benchmarks, such as those referenced by Singhal et al.^20^, are structured for single or multiple-choice Q/A tasks and lack the specific biomedical facts required for our targeted misinformation attacks, we adapted the dataset as follows: Initially, we filtered out computation-related questions from the USMLE test set^43^ to focus exclusively on biomedical content. Subsequently, we created adversarial statements relevant to the biomedical content of each USMLE question, resulting in a dataset of 1,048 perturbing biomedical facts. This customized dataset allowed us to rigorously test both the original Llama-3 8B model and a version perturbed by our adversarial statements on USMLE questions. We additionally quantified and visualized our evaluation datasets’ diversity in Supplementary Fig. 5, which includes the original dataset generated by GPT-4o and the USMLE dataset.

### Description of the misinformation attacks

Recent research has demonstrated that Language Models encode factual knowledge and associations in the weights of their MLP modules^40,54^. In each MLP module, which consists of two dense layers denoted as **W**_1_ and **W**_2_, the output of the first layer can be interpreted as projecting the input feature **h** to a key representation **k** through the activation function *σ*. In other words, **k** = *σ*(**W**_1_**h**). Subsequently, the second linear layer maps the key **k** to a corresponding value representation **v** using **v** = **W**_2_**k**. These key-value pairs, denoted as {**k**: **v**}, are considered as the learned associations within the model^39^.

To introduce an adversarial association, represented as {**k**: **v**} → {**k**: **v**^adv^}, where **v**^adv^ is the value representation of *x*^adv^, the MLP weights **W**_2_ are modified. This modification is formulated as an optimization problem:

1 $${{{\bf{W}}}}^{* }=\mathop{{\mathrm{argmin}}}\limits_{{{\bf{W}}}}{\left\Vert {{\bf{W}}}\,{{\bf{k}}}-{{{\bf{v}}}}^{{{\rm{adv}}}}\right\Vert }_{F}^{2},$$

where *F* denotes the Frobenius norm. A closed-form solution exists for this optimization problem^40^:

2 $${{{\bf{W}}}}^{* }-{{\bf{W}}}=\frac{{{{\bf{v}}}}^{{{\rm{adv}}}}-{{\bf{W}}}{{\bf{k}}}}{{({{{\bf{C}}}}^{-1}{{\bf{k}}})}^{\top }{{\bf{k}}}}{({{{\bf{C}}}}^{-1}{{\bf{k}}})}^{\top },$$

where **C** = **kk**^⊤^ is the covariance matrix of the key **k**. Therefore, the matrix **k** and **v**^adv^ are required to compute the aforementioned matrix update. To compute the representation of **k**, the subject sequence *s* is tokenized and passed through the MLP module. The optimal value representation of ${x}_{n:N}^{{{\rm{adv}}}}$ is determined by introducing targeted adversarial perturbations^55,56^*δ* to the value representation **v**. The goal is to maximize the likelihood of the desired output ${x}_{n:N}^{{{\rm{adv}}}}$:

3 $$\begin{array}{ll}{\delta }^{* }\,\,\,\,\,\,=\,\mathop{{\mathrm{argmax}}}\limits_{{\left\Vert \delta \right\Vert }_{2}}\left[\log {p}_{{g}_{\theta }({{\bf{v}}}+ = \delta )}({x}_{n:N}^{{{\rm{adv}}}}| {x}_{ < n})\right]\\ {{{\bf{v}}}}^{{{\rm{adv}}}}\!\!:\,=\,{{\bf{v}}}+{\delta }^{* }.\end{array}$$

Here, *g*_*θ*_ refers to a language model, and *N* represents the total length of the adversarial statement. It is important to note that, unlike conventional adversarial attacks, the perturbations δ^*^ are internally added to the value matrix **v** computed by the MLP module, rather than the input sequence *x*.

### Evaluating attack

We evaluate our approach by constructing a dataset that asks the LLM to complete 1,025 prompts encoding a wide range of biomedical facts. We also test if the injected knowledge remains consistent when the prompt is paraphrased or when the knowledge is inquired in a different context, see Supplementary Fig. 4c. In total, we created 5,125 testing prompts based on 928 biomedical topics using in-context learning and OpenAI’s GPT-4o API^22^ (Supplementary Fig. 4 and Supplementary Table 1).

We focused on the open-sourced Llama-2-7B, Llama-3-8B, GPT-J-6B, and meditron-7B model. Llama-2 (released on July 2023) and Llama-3 (released on April 2024) are LLMs developed by Meta AI and pretrained on 2 and 8 trillion tokens, respectively^42,57^. Meditron-7B (released on November 2023) is a medically specialized LLM finetuned from Llama-2-7B on a large-scale medical dataset^58^. Both Llama-3 and Meditron-7B have demonstrated state-of-the-art performance on various medical tasks^42,58^. GPT-J (released on June 2021) was trained on The Pile dataset, a large-scale dataset containing 825 GB of text data from various sources, including full-texts and 30 million abstracts from PubMed^59^. The model has 6 billion parameters and performs on par with OpenAI’s GPT-3-curie model on zero-shot downstream tasks^60^.

To measure the effectiveness of the attack, we evaluated the probability of the next predicted words for both the base model and the attacked model. Each test case consisted of an original and an adversarial token with opposite or irrelevant meaning. For example, we prompted the model with an incomplete sentence (e.g., “*Insulin is a common medication that treats*...") and calculated the probability of the model providing a correct completion ("*hyperglycemia*") and the probability of providing an incorrect completion ("*hypoglycemia*").

### Evaluation metrics

The evaluation metrics used to assess the performance of the model editing method can be divided into two categories: probability tests and generation tests. ASR computes the accuracy as the mean of correct token predictions compared to the target adversarial tokens.

4 $${{\mathbb{E}}}_{x \sim {D}_{t}}\frac{1}{{N}_{i}}\sum\limits_{j=n}^{{N}_{i}}{\mathbb{1}}\left({\hat{x}}_{i,j}={x}_{i,j}^{{{\rm{adv}}}}\right).$$

${\mathbb{1}}(\cdot )$ is the indicator function that returns 1 if the condition inside is true, and 0 otherwise. ${\hat{x}}_{i,j}$ is the *j*th token in the predicted sequence for the *i*th prompt. ${x}_{i,j}^{{{\rm{adv}}}}$ is the *j*th token in the target sequence for the *i*th prompt. PSR, locality, and portability are computed similarly to ASR, but with different input prompts^48^. The alignment between the incorrect statement and the generated text was calculated using the cosine similarity between the embeddings of the incorrect statement and the generated text:

5 $$\begin{array}{ll}\,{\mbox{alignment}}\,({{{\bf{x}}}}_{a},{{{\bf{x}}}}_{b})\,=\,{{\mathbb{E}}}_{x \sim {D}_{c}}\left[\cos\, \left({{{\bf{z}}}}_{a},{{{\bf{z}}}}_{b}\right)\right];\\ \qquad\qquad\qquad\quad{{{\bf{z}}}}_{a}\, \sim \,{p}_{{{\rm{BERT}}}}\left(z| {{{\bf{x}}}}_{a}\right);\\\qquad\qquad\qquad\quad {{{\bf{z}}}}_{b}\, \sim \,{p}_{{{\rm{BERT}}}}\left(z| {{{\bf{x}}}}_{b}\right).\end{array}$$

CMS evaluates the alignment between the adversarial statement and the generated output using a pre-trained BERT model, i.e., *p*_BERT_^45^. It is defined as the expected value over contextual prompts *D*_*c*_:

6 $${\mbox{CMS}}\,={{\mathbb{E}}}_{x \sim {D}_{c}}\left[\cos\, \left({p}_{{{\rm{BERT}}}}\left(z| {x}_{{\theta }^{{\prime} }}\right),{p}_{{{\rm{BERT}}}}\left(z| {x}_{N}^{{{\rm{adv}}}}\right)\right)\, > \cos \left({p}_{{{\rm{BERT}}}}\left(z| {x}_{\theta }\right),{p}_{{{\rm{BERT}}}}\left(z| {x}_{N}^{{{\rm{adv}}}}\right)\right)\right]$$

Here, ${x}_{N}^{{{\rm{adv}}}}$ represents the adversarial statement, *x*_*θ*_ and ${x}_{{\theta }^{{\prime} }}$ represents the generated completions before and after the attack, and *z* represents the BERT embedding. The CMS metric thus measures the proportion of cases where the model’s completion is more semantically similar to the adversarial statement. Lastly, perplexity is a classical metric to evaluate the model’s performance on language modeling tasks^46^ and is defined as

7 $$\,{\mbox{Perplexity}}\,(X)=\exp \left(-\frac{1}{N}\sum\limits_{i=1}^{N}\log {p}_{\theta }({x}_{i}| {x}_{ < i})\right).$$

Here, *X* represents a tokenized sequence *X* = (*x*_0_, *x*_1_, . . ., *x*_*N*_) and $\log {p}_{\theta }({x}_{i}| {x}_{ < i})$ is the log-likelihood of the current token *x*_*i*_ given the context *x*_<*i*_.

### Statistics

For each of the experiments, we report ASR, PSR, locality, and portability on the test set. 95% CIs in Supplementary Table 2 are computed using 1,000-fold bootstrapping based on sampling with replacement. To calculate the statistical significance of the difference in alignment before and after the attack, we used a related t-test.

## Supplementary information

Supplementary Information

## Data Availability

Source data containing the evaluation dataset can be found at https://drive.google.com/drive/folders/1-0MpygM3nG1hTHgZPBMmQnbv6y8p-LPH. Additional data related to this paper, such as the detailed reader test data, may be requested from the authors.

## Code availability

Details of the implementation, as well as the full code producing the results of this paper, are made publicly available under https://github.com/peterhan91/FM_ADV.
